# Childhood and adolescent predictors of leisure time physical activity during the transition from adolescence to adulthood: a population based cohort study

**DOI:** 10.1186/1479-5868-8-54

**Published:** 2011-06-01

**Authors:** Kim A Jose, Leigh Blizzard, Terry Dwyer, Charlotte McKercher, Alison J Venn

**Affiliations:** 1 Menzies Research Institute Tasmania, University of Tasmania, Private Bag 23, Hobart, Tasmania, Australia; 2 Murdoch Children's Research Institute, Royal Children's Hospital, Flemington Road, Parkville, Victoria, Australia

## Abstract

**Background:**

Few studies have investigated factors that influence physical activity behavior during the transition from adolescence to adulthood. This study explores the associations of sociodemographic, behavioral, sociocultural, attitudinal and physical factors measured in childhood and adolescence with physical activity behavior during the transition from adolescence to adulthood.

**Methods:**

Childhood and adolescent data (at ages 7-15 years) were collected as part of the 1985 Australian Health and Fitness Survey and subdivided into sociodemographics (socioeconomic status, parental education), behavioral (smoking, alcohol, sports diversity, outside school sports), sociocultural (active father, active mother, any older siblings, any younger siblings, language spoken at home), attitudinal (sports/recreational competency, self-rated health, enjoyment physical education/physical activity, not enjoying school sports) and physical (BMI, time taken to run 1.6 km, long jump) factors. Physical activity between the ages 15 and 29 years was reported retrospectively using the Historical Leisure Activity Questionnaire at follow-up in 2004-2006 by 2,048 participants in the Childhood Determinants of Adult Health Study (CDAH). Australia's physical activity recommendations for children and adults were used to categorize participants as persistently active, variably active or persistently inactive during the transition from adolescence to adulthood.

**Results:**

For females, perceived sports competency in childhood and adolescence was significantly associated with being persistently active (RR = 1.88, 95% CI = 1.39, 2.55). Smoking (RR = 0.31 CI = 0.12, 0.82) and having younger siblings (RR = 0.69 CI = 0.52, 0.93) were inversely associated with being persistently active after taking physical and attitudinal factors into account. For males, playing sport outside school (RR = 1.47 CI = 1.05, 2.08), having active fathers (RR = 1.25 CI = 1.01, 1.54) and not enjoying school sport (RR = 4.07 CI = 2.31, 7.17) were associated with being persistently active into adulthood. Time taken to complete the 1.6 km run was inversely associated with being persistently active into adulthood (RR = 0.85 CI = 0.78, 0.93) after adjusting for recreational competency.

**Conclusions:**

Perceived sports competency (females) and cardiorespiratory fitness, playing sport outside school and having active fathers (males) in childhood and adolescence were positively associated with being persistently active during the transition from adolescence to adulthood.

## Background

The health benefits of regular physical activity are well established [1] and participation in regular physical activity over time is associated with a decrease in all-cause mortality [2]. Longitudinal studies show a decline in physical activity with increasing age [3-5] with physical activity tracking at low to moderate levels across the life span [6,7]. Physical fitness measures show greater longitudinal stability than physical activity [3,6]. Studies reveal a decrease in physical activity participation during adolescence [5,8] and differences in patterns of physical activity participation for males and females [4,9].

Longitudinal studies of adolescents with repeated measures of physical activity show that only a minority were classified as persistent exercisers across the study period [10,11]. In a study of Finnish youth at ages 16, 17 and 18 only 19.1% of boys and 11.2% of girls were categorized as persistent exercisers across all three ages [10]. In a longitudinal New Zealand study only 5.8% of females and 15.8% of males were found to meet physical activity recommendations at age 15 and age 18 [11]. The transition from late adolescence to adulthood is a time of intense social transition associated with a number of significant life events. Physical activity researchers have called for further investigation of physical activity behaviors during life transitions [12].

Studies of childhood predictors of adult physical activity have investigated sociodemographic factors [13-15], sports participation [14,16], physical activity in childhood or adolescence [7,14] and physical fitness measures [3,17]. None have investigated all of these measures or focused on the transition from adolescence to adulthood. We were able to explore the association between sociodemographic, behavioral, sociocultural, attitudinal and physical factors measured in childhood and adolescence on maintaining physical activity through to young adulthood.

## Methods

### Participants

Childhood data for this study were taken from the 1985 Australian Schools Health and Fitness Survey (ASHFS), a national survey of 8,498 7-15 year old schoolchildren. Details of the 1985 sampling strategy are described elsewhere [18]. Participants underwent a range of health and fitness tests including aerobic fitness, muscular strength and anthropometry. Children aged 9 years and over (n = 6,559) also completed a questionnaire in groups of four under supervision of a trained data collector. The questionnaire asked about participation in sport and exercise, perceived competency at sport and exercise, smoking and alcohol use, parental exercise habits, and attitudes towards sport, exercise and health.

In 2001-2002, 6,840 (81%) of the ASHFS participants were located and invited to enrol in the Childhood Determinants of Adult Health (CDAH) study. A total of 5,170 (61% baseline) enrolled in the follow up study and completed a short questionnaire. Additional questionnaires, including the Historical Leisure Activity Questionnaire (HLAQ) were completed by 2,583 CDAH participants. This study included those who had completed both the childhood questionnaire and the HLAQ at follow-up (n = 2,048).

The CDAH study was approved by the Southern Tasmania Health and Medical Human Research Ethics Committee and all participants provided written informed consent.

### Physical Activity Measures

The HLAQ was developed by Kriska and colleagues to retrospectively assess lifetime participation in leisure activity [19,20]. A modified version of this questionnaire was used to capture Australian sporting and recreational activities and assess participation in leisure activity in participants from age 15 years to follow-up. Participants were asked to include activities that they had participated in with friends, an organised team, group, club or for school, but not including those done during physical education classes. The HLAQ required participants to estimate the number of years, months per year and hours per week that they participated in each activity over three age periods: 15-19, 20-24 and 25-29 years. Average minutes per week spent in activities was then calculated for each of the three age groups. Energy expenditure (metabolic equivalents) was estimated using the Ainsworth compendium of physical activities [21]. Participants were categorized as active if they met Australia's physical activity recommendations for children/adolescents (420 mins moderate to vigorous physical activity/week) at ages 15-19 or adult recommendations (150 mins moderate to vigorous physical activity/week) at ages 20-29. Persistently active participants met the guidelines at each of the three age groupings. Those who failed to meet the recommendations at any time period were classified as persistently inactive and the remainder were classified as variably active.

At follow-up participants completed the long version of the International Physical Activity Questionnaire (IPAQ) [22]. Weekly minutes of leisure time physical activity were used to indicate discretionary physical activity. Cardiorespiratory fitness was also measured using a bicycle ergometer test to estimate physical work capacity at a heart rate of 170 beats/minute (PWC170) on a Monark bicycle ergometer (model 828E, Monark Exercise AB, Sweden) using a standardized protocol [23]

### Childhood and Adolescent Measures

With the exception of parental education (reported retrospectively at follow-up) all measures were collected at baseline in 1985. Sociodemographic information collected included postcode of residence at baseline. The highest level of education achieved by parents when participants were aged 12 years was collected retrospectively at follow-up. Postcode at baseline was used to categorize participants using the Australian Bureau of Statistics Socio-economic Index for Areas (SEIFA) and 1981 census data. SEIFA is a summary of five indices measuring different aspects of socioeconomic status based on questions asked in the Australian population census. All postcodes are classified into four categories (low, medium-low, medium-high and high) using the index of relative socioeconomic disadvantage. Parental education was categorized into high (university), medium (trade/vocational) and low (school only).

Behavioral factors included frequency of smoking and alcohol use, sports played and whether the sports were played for school or a club. For analysis, smokers were defined as those who had smoked at least one cigarette in the past week, and alcohol users indicated whether they had drunk alcohol on at least 1 or 2 days in the past week. Participants were asked to list up to six sports they had played in the past year and whether they played the sport for a club, school, both or neither. For analysis, diversity of sports was dichotomised to playing three or more sports in the past year or playing less than three. Outside school sport was defined as playing sport for a club. This variable was dichotomised as playing sport outside school (yes/no).

Sociocultural information collected included language spoken at home, presence of older and younger siblings in the family and physical activity of parents. For analysis, language spoken at home was dichotomised as English or other. Information on siblings was dichotomised as any older/younger siblings (yes/no). Parental activity levels were categorized from responses to the question 'Does your mother/father exercise regularly?' Participants were provided with the examples jog, play sport, go to a gym and do aerobics. Possible responses were yes/no/don't know. The variable 'Active mother' or 'Active father' indicated which parent was active regularly.

Attitudinal factors collected included self-rated health, enjoyment of school sport, physical education (PE) and physical activity, perceived competency at each sport and perceived competency at five physical recreational activities listed (tennis, swimming 100 m, ice/roller skating, squash/badminton/racquetball, skateboarding). For analysis, self-rated health was collapsed from five levels to two (good/very good or average/poor/very poor). Enjoyment of PE was dichotomised (yes/no) from the responses enjoyed very much/quite a lot/sometimes or not much/not at all. Enjoyment of school sport was also dichotomised (yes/no) from the same response options. However, enjoyment of school sport was analysed using those who did enjoy school sport as the reference category and renamed 'Do not enjoy school sport'. Participants were asked if they enjoyed physical activity and provided with the options yes/no. For each sport the children played they were asked to indicate how good they felt they were at the sport compared to others of their age. Responses were better, same or worse. A perceived competency score was developed using the average score for the total number of sports played. This was then dichotomised, using the median score, as better than peers or same as/worse than peers. A similar method was used to create the perceived competency score for those who had participated in at least two of the physical recreation activities listed on the questionnaire.

Physical measures in childhood included time taken to complete the 1.6 km long run (measured in minutes) and the standing long jump (measured in centimetres). Anthropometric measures including height and weight were measured in childhood. From these, body mass index (BMI kg/m^2^) was calculated. Using internationally accepted age and sex specific cut-points for overweight in children participants were classified as normal weight or overweight/obese [24].

### Statistical Analysis

Childhood/adolescent predictors were analysed separately for males and females because sex differences are consistently found in physical activity participation [9]. Log multinomial regression made it possible to estimate the risk of being variably active, and the risk of being persistently active. All analysis was adjusted for age and the persistently inactive group was used as the identified category. Univariable log multinomial regression analysis of childhood predictors of physical activity persistence in early adulthood was conducted separately for males and females [25]. After checking the scale of the long jump and 1.6 km run it was decided that the relationship with log risk was approximately linear and the results have been presented with continuous variables. Variables were grouped into sociodemographic (parental education, SES code), behavioral (smoking, alcohol, outside school sports, sports diversity), sociocultural (language spoken, parental physical activity, siblings), attitudinal (perceived sports competency, perceived recreational competency, self rated health, enjoyment of school sport, physical education and physical activity) and physical (long jump, long run, BMI) factors [26].

Covariates selected for further investigation in multivariable analysis were those that were statistically significant in univariable analysis. They were retained in the final models either if they were statistically significant or if their inclusion resulted in a marked (>10%) change in the coefficients of other covariates retained in the model. The final sample size for multivariable analysis (M = 797, F = 962) excluded those who did not answer questions about competency because they had not played organised sport (n = 77) or participated in at least two of the physical recreational activities (n = 39) or did not answer the question about enjoyment of school sport because they did not have school sport (n = 93). There were also a small number of missing variables (M = 14, F = 44). Data were analysed using STATA version 10.1.

## Results

### Loss to follow-up

There were no material differences between our study participants (n = 2,048) and eligible non-participants (9 to 15 year-olds at baseline, n = 4,511) with respect to mean age at baseline, mean number of sports played or mean BMI for males or females. Non-participants were more likely to be male (53.7% compared to 46.3%), more likely to smoke (11.4% compared to 7.8%) and more likely to be classified as low SES by area of residence in childhood (10.2% compared to 7.2%).

Participants were classified as persistently active, variably active or persistently inactive across the three age groups 15-19, 20-24, 25-29 years using the HLAQ (Table 1). One fifth of males and females were categorized as persistently active (19.9%). Of those who were persistently active 60.2% were males. One third of participants were persistently inactive across the three age groupings with 67.4% of these being females. The largest grouping was the variably active group with 49.8% participants.

**Table 1:** Physical activity category during transition to adulthood

	n	Persistently Inactive n (%)	Variably Active n (%)	Persistently Active n (%)
				
**Male**	922	203 (22.0)	474 (51.4)	245 (26.6)
				
**Female**	1,126	419 (37.2)	545 (48.4)	162 (14.4)
				
**Total**	2,048	622 (30.3)	1,019 (49.8)	407 (19.9)

The validity of the HLAQ was demonstrated using data from the 2,005 CDAH participants who completed the HLAQ and physical measures at follow-up (2004-2006). Median duration of total historical activity decreased from 5.5 hours at 15-19 years to 3.2 hours at 25-29 years for males and 2.8 hours at 15-19 years to 2.3 hours at 25-29 years for females. Males reported higher durations of activity than females at each age period. Correlations between total historical activity and childhood sports participation were stronger for older than younger children. Total historical activity was significantly correlated with past-week leisure physical activity (Females *r *= 0.13 to 0.37, Males *r *= 0.14 to 0.31) and cardiorespiratory fitness (Females *r *= 0.09 to 0.29, Males *r *= 0.09 to 0.23) in adulthood (see Table 2).

**Table 2:** Spearman correlations between historical leisure activity, physical activity and cardiorespiratory fitness in childhood and adulthood

HLAQ	Childhood Measures, by age‡				Adult Measures^	
	
Total Activity†	Sport		1.6 km run		LPA_tot_	PWC170
	Younger	Older	Younger	Older		
						
**Males**						
15-19 years	0.07	0.22**	0.03	0.12^¶^	0.14**	0.09^\\^
20-24 years	0.10^¶^	0.20**	0.08	0.07	0.22**	0.13**
25-29 years	0.09^¶^	0.15^\\^	0.08	-0.03	0.31**	0.23**
						
**Females**						
15-19 years	0.09^¶^	0.23**	0.08^¶^	0.20**	0.13**	0.12**
20-24 years	0.01	0.13^¶^	0.04	0.20**	0.21**	0.19**
25-29 years	0.01	0.18**	0.07	0.21**	0.37**	0.29**

†By age period

HLAQ = Historical Leisure Activity Questionnaire; Sport = participation in school and extracurricular sport during the past week; LPA_tot _= total past-week leisure physical activity (International Physical Activity Questionnaire); PWC170 = physical working capacity at 170 beats/minute

Younger (9-12 years), Older (13-15 years)

‡Childhood Measures; Males, Younger (n = 511), Older (n = 369). Females, Younger (n = 628), Older (n = 402)

^Adult measures; Males (n = 1,057), Females (n = 1,305)

^¶^*p *< 0.05

^\\^*p *< 0.01

**p < 0.001

Additional file 1, (Tables S1 and S2) shows associations between childhood factors and physical activity category for univariable analysis. For females, diversity of sport (positive), perceived sports competency (positive), smoking (inverse), having younger siblings (inverse) and time taken to run the 1.6 km long run (inverse) were all significantly associated with being persistently active in young adulthood. Perceived recreational competency (positive), long jump distance (positive) and not enjoying school sport (inverse) were found to be associated with being variably active in young adulthood.

For males, sociodemographic status medium-high, diversity of sport, playing sport outside school, having an active father, speaking English at home, perceived sports competency, perceived recreational competency (all positive) and time taken to run the 1.6 km long run (inverse) were all significantly associated with physical activity persistence in young adulthood. Sociodemographic status medium-low (inverse) and medium-high (inverse), speaking English at home (inverse) and time taken to run the long run (positive) were associated with being variably active.

Results from the final multivariable analysis are shown in Tables 3 and 4. For females, perceived sports competency in childhood was positively associated with being persistently active. Smoking in childhood and having younger siblings were inversely associated with being persistently active. Time taken (minutes) to complete the 1.6 km run was inversely associated with being persistently active males. Playing sport outside school, having an active father and not enjoying school sport were positively associated with being persistently active males. Not enjoying school sport was inversely associated with being a variably active female and male while time taken to run 1.6 km and perceived sports competency were positively associated with being variably active males.

**Table 3:** Childhood and adolescent predictors of physical activity persistence in young adulthood, multivariable analysis females, n = 962

	Variably Active			Persistently Active		
Childhood Variables	Adj RR †	95%CI	p	Adj RR †	95%CI	p
**Behavior**						
Smoking^a^	1.05	(0.83,1.31)	0.69	0.31	(0.12,0.82)	0.02
**Physiology**						
Long jump^b^	1.00	(1.00,1.01)	0.09	0.99	(0.99,1.00)	0.10
**Sociocultural**						
Younger siblings^c^	1.10	(0.96,1.26)	0.18	0.69	(0.52,0.93)	0.02
**Attitude**						
Sports competency^d^	0.95	(0.82,1.09)	0.44	1.88	(1.39,2.55)	<0.01
Rec competency^e^	1.11	(0.97,1.27)	0.12	0.83	(0.61,1.14)	0.26
Do not enj sch sport^f^	0.42	(0.19,0.92)	0.03	0.93	(0.32,2.66)	0.89

†Adjusted for all other variables in the table

^a^Smoking, dichotomised as Yes/No

^b^Distance in the long jump (cm) continuous variable

^c^Any younger siblings, dichotomised as Yes/No

^d^Perceived sports competency compared to peers, better than peers at sport, dichotomised

^e^Rec competency = Perceived recreational competency, ability to complete a task, dichotomised

^f ^Do not enj sch sport = Do not enjoy school sport, dichotomised as Yes/No

**Table 4:** Childhood and adolescent predictors of physical activity persistence in young adulthood, multivariable analysis males, n = 797

	Variably Active			Persistently Active		
Childhood Variables	Adj RR †	95%CI	p	Adj RR †	95%CI	p
**Behavior**						
Outside school sports^a^	0.92	(0.78,1.09)	0.33	1.47	(1.05,2.08)	0.03
**Physiology**						
Longrun^b^	1.06	(1.02,1.11)	<0.01	0.85	(0.78,0.93)	<0.01
**Sociocultural**						
Active father^c^	0.96	(0.82,1.10)	0.54	1.25	(1.01,1.54)	0.04
**Attitude**						
Sports competency^d^	1.18	(1.02,1.36)	0.03	1.08	(0.86,1.35)	0.52
Rec competency^e^	0.98	(0.85,1.12)	0.73	1.23	(0.98,1.54)	0.07
Do not enj sch sport^f^	0.36	(0.13,0.98)	0.04	4.07	(2.31,7.17)	<0.01

†Adjusted for all other variables in the table

^a^Smoking, dichotomised as Yes/No

^b^Distance in the long jump (cm) continuous variable

^c^Active father, dichotomised Yes/No

^d^Perceived sports competency compared to peers, better than peers at sport, dichotomised

^e^Rec competency = Perceived recreational competency, ability to complete a task, dichotomised

^e ^Do not enj sch sport = Do not enjoy school sport, dichotomised as Yes/No

## Discussion

This is the first study to explore the association between child and adolescent demographic, behavioral, sociocultural, attitudinal, and physical factors and physical activity behavior during the transition from adolescence to young adult. Young adults were categorized as persistently active, variably active or persistently inactive during this transitional life stage. Child and adolescent factors were more strongly associated with being persistently active into adulthood than variably active. The predictors varied according to gender and also physical activity category.

In our study, perceived sports competency was significantly associated with persisting with physical activity into adulthood for females. Males who assessed themselves as better than their same-age peers in sport were a little more likely to be variably active in adulthood, but not significantly more likely to be persistently active. Perceived competency refers to an individual's assessment of his/her own competency with respect to a task. Perceived competency is related to actual competency, but incorporates socioenvironmental factors such as feedback and reinforcement from significant others [27,28]. For sports competency feedback also occurs via the outcome of participation (e.g. winning, finishing place, trophies). Assessment may be self-referenced (an individual evaluates their ability to perform the task independently of the performance of others) or other-referenced (an individual compares their ability to others) [29]. In our study children's assessments of sports competency was other-referenced while recreational competency was self-referenced.

Previous longitudinal studies have found associations between teacher assessment of a student's ability (actual competency) in school sport or physical education in adolescence and physical activity in adulthood [13,16]. Longitudinal studies in children and adolescents have shown an association between perceived sports competency and involvement in physical activity during adolescence [30,31]. To our knowledge no previous longitudinal studies have examined the effect of perceived sports competency in childhood or adolescence on physical activity participation in adulthood. The current findings suggest that perceived sports competency is a particularly important factor for persistent physical activity by females. Improving perceived sports competency can occur through teaching specific skills to increase actual competency as well as by providing opportunities and supportive environments in which to develop or practise these skills [27].

In our study measures of physical fitness were associated with physical activity persistence by males and, less strongly, by females. For every minute taken to run the 1.6 km long run males were 15% less likely to be persistently active in adulthood and 6% more likely to be variably active into adulthood. In other words, the faster they ran the 1.6 km long run (indicating higher fitness) the more likely they were to be persistently active. The few studies that have examined the association between physical fitness in adolescence and physical activity in adulthood have found a positive relationship, although contrary to our study this relationship has been strongest for females and not males. These studies used measures of maximal oxygen uptake as well as endurance runs to assess cardiorespiratory fitness [3,17]. Measures of strength were included in these studies, but they did not include the long jump and they did not incorporate measures of broad psychosocial, sociocultural or demographic factors.

Unexpectedly, not enjoying school sport was positively associated with being persistently active in males. There were only a small number of children in each physical activity category who did not enjoy school sport. The small number of boys who did not enjoy school sport but were categorized as persistently active (n = 8) were aged from 9 to 15 years. Five of them ran the long run faster than the mean for their age group suggesting they were fitter than average and rated themselves as better than their peers in sport (n = 4) and physical recreation such as swimming, tennis and skateboarding (n = 5). Enjoyment of sport can be associated with competition, social factors, skill development, competency, physical sensation and challenge or achievement [32]. Given the small number of boys in this category and their age range it is difficult to explain this finding. It is possible that the type of activities offered for school sport were not activities at which they were proficient. Girls who did not enjoy school sport were less likely to be variably active into adulthood when compared with those who did enjoy school sport. The gender differences with respect to enjoyment of school sport may reflect differing sources of sport enjoyment.

Parental influences such as support and modelling have been identified as significant for physical activity in children and adolescents [9]. In our study parental physical activity was not associated with physical activity persistence in females. Father's physical activity was significantly associated with persisting with physical activity during this transitional life stage in males. A review of parental influences on physical activity of children and adolescents found that the relationship between parental physical activity levels and child physical activity was equivocal [33]. However, the majority of these studies were cross-sectional and not population based samples. The review did find evidence to support strong correlations between father and child physical activity levels, particularly fathers and sons. In a recent longitudinal study exploring the determinants of physical activity in adolescents aged 10 to 18 years in the USA mothers' physical activity was associated with physical activity in males and females [34]. In this study fathers' physical activity was not measured.

Playing sport outside school was also found to be significant for males in our study. This positive relationship between extracurricular sports participation during childhood and adolescence and physical activity in adulthood has been found in other longitudinal studies [14,16,35,36]. Where gender differences have been investigated this relationship has been found to be stronger for males than females [35]. Involvement in sport outside the school environment during childhood and adolescence may help provide continuity during the transition from adolescence to adulthood when young people leave school.

The strong inverse relationship seen in our study between smoking and physical activity is consistent with findings from other studies that have investigated this relationship [8,37]. Clustering of health promoting or risky behaviors is common and has been found in the same population of young adults [38]. Smoking is commonly associated with more sedentary lifestyles although the mechanism for this is unclear [37]. In this study the association for males and females suggested that smokers were less likely to be persistently active, but this was statistically significant only for females. The gender difference in the relationship between smoking and physical activity have been found in other studies investigating this relationship [37].

In our study, those females who had younger siblings were significantly less likely to be physical activity persisters into young adulthood. Our data do not provide insight into this finding. It is possible that it may reflect sociocultural expectations of female children to spend time caring for younger siblings thus providing less time for participating in physical activity, but this is conjecture. Studies investigating sibling relationships and physical activity have focused on assessing the relationship between physical activity participation among siblings [39]. In one study exploring family structure and physical activity in children, data on siblings were categorized according to the presence of older siblings, total number of siblings and sex of siblings [40]. The effect of younger siblings was not explored. Those children who had an older sibling were more likely to spend time in moderate intensity physical activity. This relationship was dependent on parental status.

Using the HLAQ, we found that one-fifth of young people persisted with leisure time physical activity between the ages of 15 and 29 years. The relatively small proportion of persistently active participants is consistent with other studies that have repeated measures of physical activity during this transition period [10,11,41]. Males were more likely to be persistently active than females; this difference has also been reported previously [4,10,42]. The largest physical activity category was the variably active. The variably active category included subgroups of young people who were increasingly or decreasingly active, but not persistently. However, statistical analysis of these separate subgroups (results not shown) provided no further insights than when the variably active category was considered as a whole. There were few childhood factors associated with being variably active, and physical activity participation for this group may be more strongly influenced by current life circumstances.

A limitation of this study is the use of the HLAQ that relies on participants' recall of their physical activity when they were aged 15-29 years. The mitigating factor is that we might expect accuracy of recall to be greater for organised leisure time physical activity than casual or incidental physical activity. Furthermore, organised physical activity is commonly of higher intensity than casual physical activity and in this study classification was based on time spent in moderate to vigorous physical activity and not on the other components that sum to total physical activity. Other studies have found that recall of physical activity over ten years is reliable [43,44] and that the HLAQ is reproducible [20,45]. The HLAQ has also been found to be predictive of bone mass in postmenopausal women [20]. Measuring children's physical activity and attitudes by self-report is also challenging [46]. However, the childhood physical activity self-report measures used in this study have been shown to be positively associated with the childhood cardiorespiratory fitness measures [15].

Another potential limitation of the study is the number of participants lost to follow-up. Compared to participants, non-participants were more likely to be male, smokers and classified as low SES; the effect of this loss to follow-up is not clear. The prevalence estimates we have reported for categories of physical activity persistence and other studies factors may not be generalizable beyond this sample, therefore. More importantly, however, the analytical associations reported should be robust because we have been able to explore associations with and adjust for a wide range of study factors, and none of those factors was so limited in distribution in this sample that estimation was infeasible.

## Conclusions

This is the first study to examine the influence of a range of childhood demographic, behavioral, attitudinal, sociocultural and physical factors on physical activity behaviors during the transition from adolescence to adulthood. The HLAQ enabled categorisation of physical activity behaviors into three groups (persistently active, variably active, persistently inactive) during this significant transitional life stage. This is a time when individuals move away from the influence of their family of origin and begin to establish lifestyles and behaviors for themselves. From our large population-based sample with 20 year follow-up we showed that childhood and adolescent factors did influence physical activity behaviors during this transitional life stage. Factors varied according to gender and physical activity category.

In our study we found that perceived sports competency for females and time taken to run the long run (physical fitness) for males were found to be significant predictors of persisting with physical activity into adulthood. Sociocultural and behavioral factors such as father's physical activity and playing sport outside the school environment for males and smoking for females were also found to be predictive. These factors are all modifiable within the school or home environment. Childhood and adolescent factors were less strongly associated with being variably active into adulthood. A greater understanding of the correlates of physical activity behaviors during transitional life stages can assist in the development of interventions that aim to promote physical activity behaviors over the lifespan. From our study, interventions that focus on developing perceived sports competency and physical fitness for males and females in childhood and adolescence could be effective.

## Competing interests

The authors declare that they have no competing interests.

## Authors' contributions

KJ contributed to statistical analysis and drafting the manuscript. TD and AV contributed to study conceptualisation, design and co-ordination and drafting the manuscript. LB and CM contributed to statistical analysis and drafting the manuscript. All authors read and approved the final manuscript.

## Supplementary Material

Additional file 1 **Table S1.** Factors associated with persistence in PA during the transition from childhood to adulthood, univariable analysis males Table S2. Factors associated with persistence in PA during the transition from childhood to adulthood, univariable analysis females. Click here for file
