# Supplementary material for: Childhood and adolescent predictors of leisure time physical activity during the transition from adolescence to adulthood: a population based cohort study
Source: Int J Behav Nutr Phys Act. 2011 Jun 1;8:54. doi: 10.1186/1479-5868-8-54 (PMC3129289; doi:10.1186/1479-5868-8-54)
Supplement: Additional file 1 — Table S1. Factors associated with persistence in PA during the transition from childhood to adulthood, univariable analysis males Table S2. Factors associated with persistence in PA during the transition from childhood to adulthood, univariable analysis females. [file 1479-5868-8-54-S1.DOC]

# Additional file 1

| **Table S1. Factors associated with persistence in PA during the transition from childhood to adulthood, univariable analysis males^a^** | | | | | | | | | |
| --- | --- | --- | --- | --- | --- | --- | --- | --- | --- |
| **Childhood** | **Persistently Inactive** |  | **Variably Active** | |  |  | **Persistently Active** | |  |
| **Variables** | % or  mean |  | % or mean | RRª (95%CI) | p value |  | % or mean | RRª (95%CI) | p value |
| **Demographic** |  |  |  |  |  |  |  |  |  |
| Parental education | |  |  |  |  |  |  |  |  |
| Low(n=363) | 24.5 |  | 50.7 | 1.0 (ref) |  |  | 24.8 | 1.0 (ref) |  |
| Medium(n= 303) | 20.8 |  | 52.8 | 1.03(0.89,1.19) | 0.71 |  | 26.4 | 1.09(0.84,1.42) | 0.50 |
| High(n=256) | 19.9 |  | 50.8 | 0.99(0.85,1.16) | 0.95 |  | 29.3 | 1.20(0.92,1.55) | 0.17 |
| SES (Area of residence) | |  |  |  |  |  |  |  |  |
| Low(n=78) | 19.2 |  | 62.8 | 1.0 (ref) |  |  | 18.0 | 1.0 (ref) |  |
| Med-low(n=319) | 24.1 |  | 49.5 | 0.76(0.62,0.93) | 0.01 |  | 26.3 | 1.55(0.93,2.58) | 0.09 |
| Med-high(n=244) | 22.1 |  | 48.4 | 0.74(0.59,0.91) | 0.01 |  | 29.5 | 1.77(1.06,2.96) | 0.03 |
| High(n=242) | 17.8 |  | 54.1 | 0.83(0.67,1.02) | 0.08 |  | 28.1 | 1.67(1.00,2.80) | 0.05 |
|  |  |  |  |  |  |  |  |  |  |
| **Behavioral** |  |  |  |  |  |  |  |  |  |
| Diversity sports | |  |  |  |  |  |  |  |  |
| <3(n=474) | 24.7 |  | 52.7 | 1.0 (ref) |  |  | 22.6 | 1.0 (ref) |  |
| ≥3(n=448) | 19.2 |  | 50.0 | 0.95(0.84,1.08) | 0.44 |  | 30.8 | 1.36(1.09,1.68) | 0.01 |
| Outside school sports | |  |  |  |  |  |  |  |  |
| No(n=275) | 28.0 |  | 51.6 | 1.0 (ref) |  |  | 20.4 | 1.0 (ref) |  |
| Yes(n=647) | 19.5 |  | 51.3 | 0.99(0.87,1.14) | 0.92 |  | 29.2 | 1.44(1.11,1.87) | 0.01 |
| Smoking |  |  |  |  |  |  |  |  |  |
| No(n=857) | 21.7 |  | 51.6 | 1.0 (ref) |  |  | 26.7 | 1.0 (ref) |  |
| Yes(n=65) | 26.2 |  | 49.2 | 1.00(0.77,1.30) | 0.99 |  | 24.6 | 0.84(0.54,1.32) | 0.45 |
| Alcohol |  |  |  |  |  |  |  |  |  |
| No(n=845) | 22.4 |  | 51.2 | 1.0 (ref) |  |  | 26.4 | 1.0 (ref) |  |
| Yes(n=77) | 18.2 |  | 53.2 | 1.04(0.84,1.30) | 0.69 |  | 28.6 | 1.07(0.74,1.54) | 0.72 |
|  |  |  |  |  |  |  |  |  |  |
| **Sociocultural** |  |  |  |  |  |  |  |  |  |
| Active Father | |  |  |  |  |  |  |  |  |
| No(n=630) | 23.0 |  | 52.9 | 1.0 (ref) |  |  | 24.1 | 1.0 (ref) |  |
| Yes(n=292) | 19.9 |  | 48.3 | 0.91(0.79,1.04) | 0.18 |  | 31.8 | 1.33(1.07,1.65) | <0.01 |
| Active Mother | |  |  |  |  |  |  |  |  |
| No(n=576) | 22.0 |  | 53.5 | 1.0 (ref) |  |  | 24.5 | 1.0 (ref) |  |
| Yes(n=346) | 21.9 |  | 48.0 | 0.89(0.78,1.02) | 0.10 |  | 30.1 | 1.23(0.99,1.52) | 0.06 |
| Any older siblings | |  |  |  |  |  |  |  |  |
| No(n=400) | 21.7 |  | 48.5 | 1.0 (ref) |  |  | 29.8 | 1.0 (ref) |  |
| Yes(n=522) | 22.2 |  | 53.6 | 1.09(0.96,1.24) | 0.18 |  | 24.1 | 0.83(0.67,1.03) | 0.09 |
| Any younger siblings | |  |  |  |  |  |  |  |  |
| No(n=357) | 22.1 |  | 54.1 | 1.0 (ref) |  |  | 23.8 | 1.0 (ref) |  |
| Yes(n=565) | 22.0 |  | 49.7 | 0.93(0.82,1.06) | 0.26 |  | 28.3 | 1.16(0.93,1.46) | 0.19 |
| Language spoken at home | |  |  |  |  |  |  |  |  |
| Other(n=114) | 24.6 |  | 59.6 | 1.0 (ref) |  |  | 15.8 | 1.0 (ref) |  |
| English(n=808) | 21.7 |  | 50.2 | 0.83(0.70,0.98) | 0.03 |  | 28.1 | 1.83(1.18,2.83) | 0.01 |
|  |  |  |  |  |  |  |  |  |  |
| **Attitudes** |  |  |  |  |  |  |  |  |  |
| Sports competency† | |  |  |  |  |  |  |  |  |
| Did not play(n=60) | 30.0 |  | 53.3 | excluded |  |  | 16.7 | excluded |  |
| Same/worse peers |  |  |  |  |  |  |  |  |  |
| (n=435) | 26.90 |  | 49.7 | 1.0 (ref) |  |  | 23.4 | 1.0 (ref) |  |
| Better peers(n=427) | 15.90 |  | 52.9 | 1.07(0.94,1.22) | 0.28 |  | 31.2 | 1.31(1.05,1.63) | 0.02 |
| Recreational competency † | |  |  |  |  |  |  |  |  |
| Had not tried(n=39) | 25.6 |  | 56.4 | excluded |  |  | 18.0 | excluded |  |
| Av/not do(n=458) | 25.3 |  | 51.1 | 1.0 (ref) |  |  | 23.6 | 1.0 (ref) |  |
| Very well(n=425) | 18.1 |  | 51.3 | 1.00(0.88,1.14) | 0.95 |  | 30.6 | 1.30(1.04,1.61) | 0.02 |
| Enjoyment PE† | |  |  |  |  |  |  |  |  |
| Did not do(n=52) | 34.6 |  | 42.3 | excluded |  |  | 23.1 | excluded |  |
| No(n=39) | 23.1 |  | 51.3 | 1.0 (ref) |  |  | 25.6 | 1.0 (ref) |  |
| Yes(n=811) | 21.0 |  | 52.0 | 1.04(0.76,1.42) | 0.81 |  | 27.0 | 1.01(0.58,1.74) | 0.98 |
| Do not enjoy school sport† | |  |  |  |  |  |  |  |  |
| Did not have(n=54) | 33.3 |  | 51.9 | excluded |  |  | 14.8 | excluded |  |
| No(n=832) | 21.3 |  | 51.6 | 1.0 (ref) |  |  | 27.1 | 1.0 (ref) |  |
| Yes(n=18) | 16.7 |  | 38.9 | 0.77(0.43,1.37) | 0.37 |  | 44.4 | 1.57(0.93,2.66) | 0.09 |
| Enjoyment PA | |  |  |  |  |  |  |  |  |
| No(n=31) | 45.1 |  | 32.3 | 1.0 (ref) |  |  | 22.6 | 1.0 (ref) |  |
| Yes(n=870) | 21.1 |  | 52.0 | 1.59(0.95,2.67) | 0.08 |  | 26.9 | 1.21(0.63,2.35) | 0.57 |
| Self rated health | |  |  |  |  |  |  |  |  |
| Av/ below(n=162) | 22.2 |  | 51.9 | 1.0 (ref) |  |  | 25.9 | 1.0 (ref) |  |
| Good/better(n=744) | 21.8 |  | 51.3 | 0.99(0.84,1.17) | 0.95 |  | 26.9 | 1.03(0.77,1.36) | 0.86 |
|  |  |  |  |  |  |  |  |  |  |
| **Physiological** |  |  |  |  |  |  |  |  |  |
| Long run, mins(n=872)^b^ | 8.14 |  | 8.17 | 1.05(1.00,1.09) | 0.04 |  | 7.8 | 0.86(0.78, 0.95) | <0.01 |
| BMI, kg/m² |  |  |  |  |  |  |  |  |  |
| Normal(n=841) | 21.8 |  | 51.4 | 1.0 (ref) |  |  | 26.9 | 1.0 (ref) |  |
| Ovwght/obese(n=81) | 24.7 |  | 51.9 | 1.01(0.81,1.25) | 0.95 |  | 23.5 | 0.88(0.58,1.32) | 0.54 |
| Long jump, cm(n=921)^c^  159.2 | |  | 161.1 | 1.00(0.99,1.00) | 0.74 |  | 166.0 | 1.00(0.99,1.01) | 0.11 |
| Abbreviations: PA = physical activity RR = relative risk; CI = confidence interval; ref = referent; med = medium; PE = physical education; V.good = Very good; Ovwght = Overweight | | | | | | | | |  |
|  |
| ªAdjusted for age at baseline | |  |  |  |  |  |  |  |  |
| ^b^25th, 50th, 75th percentile = 7.18, 7.78, 8.73 mins respectively | | | | | | | | | |
| ^c^25th, 50^th^, 75^th^ percentile = 142.0, 160.0, 180.0 cm respectively | | | | | | | | | |
| † Those who did not play sport, try at least two physical recreation activities or participate in PE or school sport | | | | | | | | | |
| excluded from analysis | |  |  |  |  |  |  |  |  |

| **Table S2. Factors associated with persistence in PA during the transition from childhood to adulthood, univariable analysis females^a^** | | | | | | | | |  |
| --- | --- | --- | --- | --- | --- | --- | --- | --- | --- |
| **Childhood** | **Persistently Inactive** |  | **Variably Active** | |  |  | **Persistently Active** | |  |
| **Variables** | % or  mean |  | % or mean | RRª (95%CI) | p value |  | % or mean | RRª (95%CI) | p value |
| **Demographic** |  |  |  |  |  |  |  |  |  |
| Parental education | |  |  |  |  |  |  |  |  |
| Low(n=439) | 38.3 |  | 47.6 | 1.0 (ref) |  |  | 14.1 | 1.0 (ref) |  |
| Med(n=390) | 41.5 |  | 46.4 | 0.98(0.85,1.13) | 0.76 |  | 12.1 | 0.85(0.60,1.21) | 0.36 |
| High(n=297) | 30.0 |  | 52.2 | 1.10(0.95,1.27) | 0.22 |  | 17.8 | 1.19(0.85,1.67) | 0.31 |
| SES (Area of residence) | |  |  |  |  |  |  |  |  |
| Low(n=64) | 51.6 |  | 37.5 | 1.0 (ref) |  |  | 10.9 | 1.0 (ref) |  |
| Med-low(n=414) | 35.7 |  | 50.5 | 1.35(0.97,1.88) | 0.08 |  | 13.8 | 1.17(0.56, 2.45) | 0.68 |
| Med-high(n=309) | 37.5 |  | 47.9 | 1.28(0.92,1.80) | 0.15 |  | 14.6 | 1.20(0.56,2.55) | 0.64 |
| High(n=303) | 35.0 |  | 48.8 | 1.31(0.93,1.84) | 0.12 |  | 16.2 | 1.35(0.64,2.84) | 0.44 |
|  |  |  |  |  |  |  |  |  |  |
| **Behavioral** |  |  |  |  |  |  |  |  |  |
| Diversity sports played | |  |  |  |  |  |  |  |  |
| <3(n=630) | 40.3 |  | 47.3 | 1.0 (ref) |  |  | 12.4 | 1.0 (ref) |  |
| ≥3(n=496) | 33.3 |  | 49.8 | 1.05(0.93,1.19) | 0.42 |  | 16.9 | 1.36(1.03,1.81) | 0.03 |
| Outside school sports | |  |  |  |  |  |  |  |  |
| No(n=437) | 40.0 |  | 46.7 | 1.0 (ref) |  |  | 13.3 | 1.0 (ref) |  |
| Yes(n=689) | 35.4 |  | 49.5 | 1.06(0.94,1.2) | 0.34 |  | 15.1 | 1.18(0.88,1.59) | 0.27 |
| Smoking |  |  |  |  |  |  |  |  |  |
| No(n=1031) | 36.4 |  | 48.4 | 1.0 (ref) |  |  | 15.2 | 1.0 (ref) |  |
| Yes(n=95) | 46.3 |  | 48.4 | 1.01(0.80,1.26) | 0.96 |  | 5.3 | 0.39(0.16,0.94) | 0.04 |
| Alcohol |  |  |  |  |  |  |  |  |  |
| No(n=1064) | 36.8 |  | 48.8 | 1.0 (ref) |  |  | 14.4 | 1.0 (ref) |  |
| Yes(n=62) | 43.6 |  | 41.9 | 0.86(0.64,1.16) | 0.33 |  | 14.5 | 1.10(0.59,2.03) | 0.78 |
|  |  |  |  |  |  |  |  |  |  |
| **Sociocultural** |  |  |  |  |  |  |  |  |  |
| Active Father | |  |  |  |  |  |  |  |  |
| No(n=706) | 38.8 |  | 47.7 | 1.0 (ref) |  |  | 13.5 | 1.0 (ref) |  |
| Yes(n=420) | 34.5 |  | 49.5 | 1.04(0.91,1.17) | 0.57 |  | 16.0 | 1.15(0.86,1.54) | 0.33 |
| Active Mother | |  |  |  |  |  |  |  |  |
| No (n=671) | 39.3 |  | 47.2 | 1.0 (ref) |  |  | 13.4 | 1.0 (ref) |  |
| Yes (n=455) | 34.1 |  | 50.1 | 1.06(0.94,1.20) | 0.35 |  | 15.8 | 1.16(0.87,1.54) | 0.30 |
| Any older siblings | |  |  |  |  |  |  |  |  |
| No(n=462) | 39.4 |  | 47.6 | 1.0 (ref) |  |  | 13.0 | 1.0 (ref) |  |
| Yes(n=664) | 35.7 |  | 48.9 | 1.04(0.91,1.16) | 0.66 |  | 15.4 | 1.16(0.86,1.55) | 0.34 |
| Any younger siblings | |  |  |  |  |  |  |  |  |
| No(n=456) | 36.6 |  | 46.3 | 1.0 (ref) |  |  | 17.1 | 1.0 (ref) |  |
| Yes(n=670) | 37.6 |  | 49.9 | 1.08(0.95,1.22) | 0.23 |  | 12.5 | 0.73(0.55,0.97) | 0.03 |
| Language spoken |  |  |  |  |  |  |  |  |  |
| Other(n=120) | 39.2 |  | 50.0 | 1.0 (ref) |  |  | 10.8 | 1.0 (ref) |  |
| English(n=1006) | 37.0 |  | 48.2 | 0.96(0.80,1.17) | 0.71 |  | 14.8 | 1.32(0.78,2.26) | 0.30 |
|  |  |  |  |  |  |  |  |  |  |
| **Attitudes** |  |  |  |  |  |  |  |  |  |
| Sports competency† | |  |  |  |  |  |  |  |  |
| Did not play(n=84) | 36.0 |  | 52.0 | excluded |  |  | 12.0 | excluded |  |
| Same/worse peers  (n=670) | 40.0 |  | 48.1 | 1.0 (ref) |  |  | 11.9 | 1.0 (ref) |  |
| Better peers(n=372) | 32.5 |  | 48.1 | 1.00(0.88,1.14) | 1.00 |  | 19.4 | 1.60(1.19,2.14) | <0.01 |
| Recreational competency † | |  |  |  |  |  |  |  |  |
| Had not tried(n=43) | 35.0 |  | 58.0 | excluded |  |  | 7.0 | excluded |  |
| Av/not do(n=718) | 38.9 |  | 45.8 | 1.0 (ref) |  |  | 15.3 | 1.0 (ref) |  |
| Very well(n=365) | 34.3 |  | 52.3 | 1.15(1.01,1.30) | 0.03 |  | 13.4 | 0.84(0.62,1.15) | 0.27 |
| Enjoyment PE† |  |  |  |  |  |  |  |  |  |
| Did not do(n=54) | 35.2 |  | 57.4 | excluded |  |  | 7.4 | excluded |  |
| No(n=36) | 47.2 |  | 47.2 | 1.0 (ref) |  |  | 5.6 | 1.0 (ref) |  |
| Yes(n=1071) | 37.2 |  | 47.8 | 1.01(0.71,1.44) | 0.95 |  | 15.0 | 2.80(0.72,10.83) | 0.14 |
| Do not enjoy school sport† | |  |  |  |  |  |  |  |  |
| Did not have(n=67) | 32.9 |  | 53.7 | excluded |  |  | 13.4 | excluded |  |
| No(n=999) | 36.7 |  | 49.0 | 1.0 (ref) |  |  | 14.3 | 1.0 (ref) |  |
| Yes(n=40) | 62.5 |  | 20.0 | 0.41(0.22,0.76) | 0.01 |  | 17.5 | 1.26(0.63,2.50) | 0.51 |
| Enjoyment PA |  |  |  |  |  |  |  |  |  |
| No(n=23) | 60.9 |  | 26.1 | 1.0 (ref) |  |  | 13.0 | 1.0 (ref) |  |
| Yes(n=1073) | 37.1 |  | 48.6 | 1.87(0.94,3.71) | 0.08 |  | 14.3 | 1.16(0.40,3.35) | 0.79 |
| Self rated health |  |  |  |  |  |  |  |  |  |
| Av/below(n=205) | 42.4 |  | 44.4 | 1.0 (ref) |  |  | 13.2 | 1.0 (ref) |  |
| Good/V.Good(n=903) | 36.2 |  | 49.1 | 1.10(0.94,1.31) | 0.24 |  | 14.7 | 1.09(0.74,1.59) | 0.68 |
|  |  |  |  |  |  |  |  |  |  |
| **Physiological** |  |  |  |  |  |  |  |  |  |
| Long run,mins(1033)^b^ | 9.9 |  | 9.69 | 1.00(0.96,1.05) | 0.37 |  | 9.46 | 0.87(0.79,0.97) | 0.01 |
| BMI, kg/m² |  |  |  |  |  |  |  |  |  |
| Normal(n=1028) | 37.1 |  | 48.4 | 1.0 (ref) |  |  | 14.5 | 1.0 (ref) |  |
| Ovwght/obese(n=97) | 39.2 |  | 47.4 | 0.98(0.79,1.22) | 0.85 |  | 13.4 | 0.92(0.54,1.55) | 0.76 |
| Long jump,cm(n=1119)^c^ | 143.3 |  | 146.3 | 1.004(1.001,1.01) | 0.01 |  | 144.1 | 1.01(0.99,1.01) | 0.61 |
| Abbreviations: PA = physical activity; RR = relative risk; CI = confidence interval; ref = referent; med = medium; PE = physical education; V.good = Very good; Ovwght = Overweight  ^a^Adjusted for age at baseline | | | | | | | | |  |
| ^b^25th, 50th, 75th percentile = 8.58, 9.57, 10.67 mins | | | | |  |  |  |  |  |
| ^c^25th, 50th, 75th percentile = 130.5, 145.5, 159.0 cm | | | | |  |  |  |  |  |
| † Those who did not play sport, try at least two physical recreation activities or participate in PE or school sport | | | | | | | | | |
| excluded from analysis | | | | | | | | | |
|  |  |  |  |  |  |  |  |  |  |
